# Isoflurane Preconditioning Attenuates Brain Injury Induced by Electromagnetic Pulse via the TLR4/NF*κ*B Signaling Pathway

**DOI:** 10.1155/2019/9653494

**Published:** 2019-01-06

**Authors:** Jiang-Jing Li, Bin Deng, Xia-Jing Zhang, Miao-Miao Lv, Hui Zhao, Jin Wang, Ji-Dong Liu, Rui-Li Han, Xu-De Sun

**Affiliations:** ^1^Department of Anesthesiology, The Second Affiliated Hospital of Air Force Medical University, Xi'an 710038, China; ^2^Department of Anesthesiology, Xiang'an Hospital of Xiamen University, Xiamen 361101, China; ^3^Department of Anesthesiology, Fourth Hospital of Xi'an, Xi'an 710038, China; ^4^Department of Anesthesiology, The 323 Hospital of PLA, Xi'an 710054, China; ^5^Department of Preventive Medicine, Air Force Medical University, Xi'an 710038, China; ^6^Outpatient Department, Ministry of Foreign Affairs of the People's Republic of China, Beijing 100701, China

## Abstract

Electromagnetic pulse (EMP) is a unique type of electromagnetic radiation, and EMP exposure causes a series of biological effects. The nervous system is sensitive to EMP. We studied the neuroprotective effects of isoflurane preconditioning against EMP exposure and used hematoxylin-eosin staining (HE) to observe the effects of electromagnetic pulse and isoflurane preconditioning on neurons. Inflammatory cytokines were detected by enzyme-linked immunosorbent assay (ELISA). Western blotting was used to detect the expression of caspase-3, CD11b, TLR4, and NF*κ*Bp65. We found that after EMP exposure, the number of abnormal neurons had increased, and the expression of caspase-3, CD11b, TLR4, and NF*κ*Bp65 had also increased. Isoflurane preconditioning can reverse the above phenomenon. Moreover, we found that isoflurane preconditioning can reduce neuronal apoptosis and improve cognitive impairment induced by EMP. These findings indicate that isoflurane preconditioning can protect neurons in the cerebral cortex from EMP exposure, alleviate the inflammatory reaction and cell apoptosis, and improve cognitive impairment induced by EMP. These effects may occur through the downregulation of the TLR4/NF*κ*B signaling pathway and the inhibition of microglial activation.

## 1. Introduction

With the progress of human material civilization and the rapid development of high level, new technology, many electronic facilities and types of radio equipment produce electromagnetic radiation. The biological effects of this electromagnetic radiation have aroused concern, and relevant studies have confirmed that certain types of electromagnetic pulse radiation have detrimental effects on the organism. Because the central nervous system (CNS) is very sensitive to electromagnetic pulse radiation, tissue and organ damage can occur, and nerve behavior disorder in the CNS may be seen after exposure [1–3]. Existing studies have found that EMP exposure can lead to long-term cognitive learning changes in rats, increasing the accumulation of amyloid beta in the brain [4]. Electromagnetic pulses can induce microglial activation and change the levels of inflammatory cytokines [5]. Previous studies found that EMP can cause brain damage by inducing neuronal oxidative stress and apoptosis [6]. However, research on methods for protection against electromagnetic pulse has mostly examined physical protection [7], and the research on drug protection and treatment is scarce.

Isoflurane is one of the most common inhaled anesthetics [8]. Many studies have shown that isoflurane has a protective effect on multiple organs in the model of ischemia-reperfusion injury [9–11]. The neuroprotective effect of isoflurane preconditioning has also been verified several times [12, 13], but its neuroprotective mechanism remains unclear. In the middle cerebral arterial occlusion (MCAO) model, isoflurane preconditioning achieves neuroprotective effects by inhibiting the activation of microglial cells and reducing the secretion of inflammatory factors [14].

TLR signaling pathways induce inflammation by activating and releasing inflammatory corpuscle; recently, many studies have shown that the release of inflammatory corpuscle is associated with a variety of CNS lesions, such as infection, autoimmune disease, and injury [15]. TLR4 is a member of the toll-like receptor family, which is associated with a variety of pathological changes in central nervous system cells [16]. TLR4/NF*κ*B is the classic pathway of inflammatory response; many studies have found that TLR4 activation results in a cascade amplification effect, NF*κ*B activation, then an increase in the proinflammatory factor and stress reaction medium expression, such as TNF-*α*, cox-2, and iNOS, and these cytokines are involved in CNS diseases [17–19].

Isoflurane preconditioning was found to reduce microglial activation and the inflammatory response through the TLR4/NF*κ*B signaling pathway in a cerebral ischemia-reperfusion injury model and to reduce brain injury [14]. This study was designed to investigate the protective effect of isoflurane preconditioning on EMP brain injury and to determine whether its mechanism is mediated by the TLR4/NF*κ*B signaling pathway.

## 2. Materials and Methods

### 2.1. Experimental Protocols

#### 2.1.1. Experiment 1

Healthy adult male SD rats were randomly divided into the control group (the rats were placed in the EMP exposure space but not radiated) and the EMP exposure groups, which were divided into 3 groups: the 1 h after EMP group, 6 h after EMP group, and 24 h after EMP group. HE staining was performed to observe the effects of EMP on cerebral cortex neurons. Western blotting and immunofluorescence were used to detect the expression of CD11b, TLR4, and NF*κ*Bp65.

#### 2.1.2. Experiment 2

To study the neuroprotective effects of isoflurane preconditioning on EMP exposure, SD rats were randomly divided into 4 groups: the control group, 6 h after EMP group (EMP group, the time point according to experiment 1), EMP exposure after isoflurane preconditioning group (EMP + Iso group), and isoflurane preconditioning group (Iso group). The test items were the same as those in experiment 1.

### 2.2. Animals

Healthy adult male SD rats, weighing 200~250 g, with free access to food and water, were purchased from the animal experimental center of The Air Force Medical University.

### 2.3. EMP Exposure

The EMP (peak intensity 400 kv/m, rise time 10 ns, pulse width 350 ns, 0.5 pps, 1 Hz, 200 pulses total) would irradiate for 3 days, then the correlation tests were carried out. The same duration of EMP exposure was given every day. Each SD rat was kept in a 20 cm × 8 cm × 8 cm transparent plastic box. The rats were placed in the irradiated space, and the rats were awake.

### 2.4. Isoflurane Preconditioning

The rats were placed in a closed circulating transparent plexiglass chamber (43 cm × 32 cm × 16 cm). The gas inlet of the glass box was connected with an isoflurane volatile tank connected with oxygen, and the gas outlet was connected with a Vamos gas concentration monitor. The glass surface of the bottom of the box spread into the amount of soda lime, covered with a suitable size thin breathable sponge pad, and some padding was placed on the cotton pad to prevent rats from eating soda lime or aspiration of dust. By adjusting the oxygen flow rate and the isoflurane inhalation concentration, the gas concentration monitor showed that the outlet concentration of the glass box was 2%, so the isoflurane pretreatment lasted for 2 h and EMP exposure after the pretreatment 24 h. During isoflurane preconditioning, limb movements, respiratory movements, and loss of consciousness were observed closely in rats.

### 2.5. HE Staining

The morphology of neurons after exposure to EMP was observed by HE staining. The rats were perfused with 4% (*w*/*v*) paraformaldehyde. The brain tissues were removed, dehydrated, paraffin embedded, and then sliced (4 *μ*m). The tissue slices were baked on the baking machine at approximately 50°C for 1 h. After xylene transparent (xylene I 15 min and xylene II 15 min) dehydration in gradient alcohol (100% ethanol I 5 min, 100% ethanol II 5 min, and 80% ethanol 5 min), the slices were placed in distilled water for 5 min. Then, the slices were stained in hematoxylin semen staining solution for 5 min, rinsed fully with tap water for 5 min, placed in an alcohol hydrochloride differentiation fluid, and removed rapidly. Next, the slices were flushed with tap water for approximately 30 s and then eosin stain dyed for 1 min. After removal, they were washed with distilled water for 20 s and needed gradient alcohol decolonization (80% ethanol slightly washed 30 s, 95% ethanol I 1 min, 95% ethanol II 1 min, anhydrous ethanol I 3 min, and anhydrous ethanol II 3 min). Finally, the slices were bathed in xylene (xylene I 3 min and xylene II 3 min) and sealed with neutral gum.

### 2.6. Enzyme-Linked Immunosorbent Assay (ELISA)

The rats were anesthetized deeply and decapitated, and the cortex was separated and stored in the refrigerator at −80°C. The frozen tissues were removed and homogenized at low temperature. After centrifugation, the supernatant was taken as the sample to be reserved. TNF-*α* and IL-1*β* were analyzed by ELISA per the manufacturer's instructions.

### 2.7. Western Blotting

Western blotting analysis was used to detect the expression of active caspase-3, CD11b, TLR4, and NF*κ*Bp65. The frozen cortical tissue was taken from the −80°C fridge and homogenized at low temperature, and the protein concentration was quantified using a BCA kit. The following primary antibodies were used: rabbit anti-caspase-3 (1 : 500; Abcam, Cambridge, UK), rabbit anti-CD11b (1 : 500; Abcam, Cambridge, UK), mouse anti-TLR4 (1 : 500; Abcam, Cambridge, UK), rabbit anti-NF*κ*Bp65 (1 : 1000; Abcam, Cambridge, UK), and mouse anti-*β*-actin (1 : 1000; CMCTAG, Milwaukee, USA). Secondary horseradish peroxidase- (HRP-) conjugated goat anti-rabbit antibody or goat anti-mouse antibody (1 : 5000 dilution) was used.

### 2.8. TUNEL Staining

To determine whether IP reduced apoptosis, TUNEL staining was performed using an In Situ Cell Death Detection Kit (Roche Diagnostics, Mannheim, Germany). The tissue sections in every group were fixed in 4% (*v*/*v*) ice-cold paraformaldehyde for 1 h, washed in phosphate-buffered solution (PBS) (0.1 M, pH 7.4) for 5 min, treated with 0.3% (*v*/*v*) H_2_O_2_ for 10 min, rinsed in PBS for 5 min, and incubated in a TUNEL reaction mixture for 1 h at 37°C (after which, the neurons were stained with DAPI for 5 min at room temperature). Images were obtained using a microscope (BX60, Olympus). Five sections from each rat were selected randomly, and neurons from five separate fields on each coverslip were counted. The average number of positive cells was counted for each individual rat.

### 2.9. Immunofluorescence Staining

Immunofluorescence staining was used to observe the expression of TLR4 and NF*κ*Bp65 in the cortex of rats. The tissue sections were dewaxed and dehydrated, such as those mentioned in the HE staining. Citrate buffer was used to repair the antigen, and then it was cooled at room temperature after the completion of the repair. Then, 3% hydrogen peroxide- (H_2_O_2_-) deionized water was added to the tissue surface and incubated at room temperature for 15 min to eliminate the activity of endogenous peroxidases. The tissue sections were cleaned with PBS and blocked with normal goat serum for 20 min. Next, the sections were added to the mouse anti-TLR4 (1 : 100) or rabbit anti-NF*κ*Bp65 (1 : 100) at 4°C overnight. Then, the sections were incubated with secondary antibody (1 : 200) accordingly for 2 h in the dark at room temperature. After nuclear DAPI staining for 10 min, the sections were sealed with an antifluorescence quenching agent.

### 2.10. Behavioral Tests

We assessed long-term memory using the Morris water-maze (MWM) test, and learning was assessed using the T-maze. The MWM test was introduced in our previous study [6]. Spatial working memory and reference memory were evaluated using the T-maze. One arm of the maze was designated as the correct arm, and the other arm was designated as the incorrect arm. The mice were placed at the end of the box and given a 5 s foot shock; the shock was continued until the mouse entered the correct arm. The mice were trained until they demonstrated avoidance; that is, they avoided the shock by entering the correct arm. The number of trails it took the mice to first demonstrate avoidance and the number of trials it took to demonstrate avoidance five times out of six continuous trials after one week were measured.

### 2.11. Statistical Analysis

SPSS 22.0 software was used to conduct the statistical analysis. All the values are presented as the means ± standard error of the mean (SEM) and were analyzed by one-factor analysis of variance (ANOVA). *P* < 0.05 was considered statistically significant.

## 3. Results

### 3.1. Effects of EMP on Neurons in the Cerebral Cortex of SD Rats

**Figure 1:**
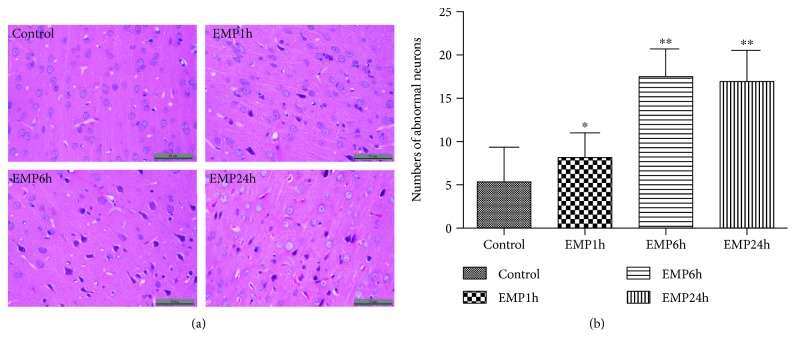
(a) Prefrontal cortex of rats with HE staining (scale bar = 50 *μ*m). The neurons of the control group were neatly arranged; however, the neurons in the EMP1h, EMP6h, and EMP24h groups exhibited cell body shrinkage and nucleus and cytoplasm deep staining. (b) Abnormal neuron number after EMP exposure (^∗^ *P* < 0.05 and ^∗∗^ *P* < 0.01 vs. the control group).

HE staining was used to observe the effect of EMP irradiation on the morphology of frontal cortex neurons. In the frontal cortex region, 5 nonrepeated regions were randomly selected, and the number of abnormal neurons in the region was calculated. After calculating the average number, the samples from each group were statistically analyzed. The results showed that after EMP irradiation, the number of abnormal neurons had increased, the cell arrangement was disordered, the cell bodies were pyknosis, and the nucleus and cytoplasm were deeply stained. The number of abnormal neurons had increased after EMP exposure (*P* < 0.05), and it was increased significantly at 6 h and 24 h after EMP exposure (*P* < 0.01) (Figure 1).

### 3.2. Effects of EMP on Caspase-3 in the Cerebral Cortex of SD Rats

**Figure 2:**
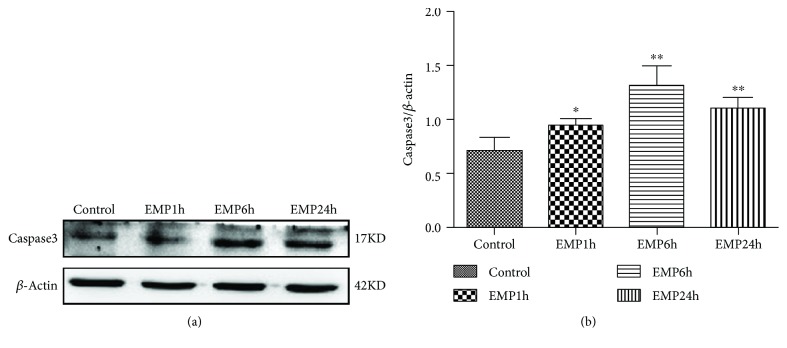
(a) Western blot of active caspase-3 after EMP exposure. (b) Quantification of relative changes in active caspase-3 expression (^∗^ *P* < 0.05 and ^∗∗^ *P* < 0.01 vs. the control group).

Caspase-3 expression in the cerebral cortex was detected by a Western blotting semi-quantitative analysis, which showed that the expression of caspase-3 in the cerebral cortex of rats in each experimental group was significantly higher than that in the control group after EMP exposure, and the difference was statistically significant (*P* < 0.05); the expression was increased significantly during the 6 h (EMP6h) and 24 h (EMP24h) after EMP exposure (*P* < 0.01) (Figure 2).

### 3.3. Effects of EMP on Microglia in the Cerebral Cortex of SD Rats

**Figure 3:**
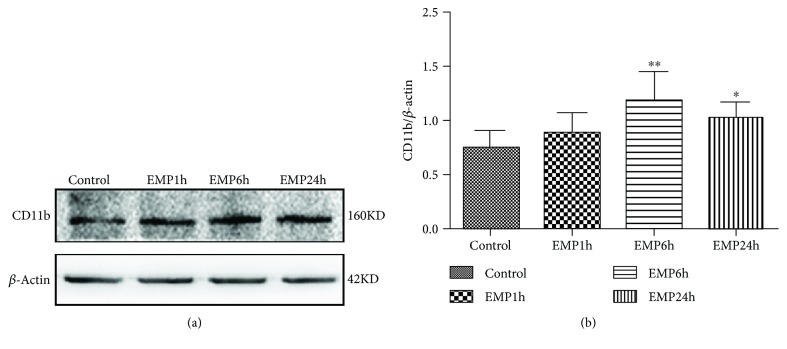
(a) Western blot of active CD11b after EMP exposure. (b) Quantification of relative changes in active CD11b expression (^∗^ *P* < 0.05 and ^∗∗^ *P* < 0.01 vs. the control group).

The changes in CD11b expression in the cerebral cortex were detected by Western blotting semiquantitative analysis, which showed that after EMP exposure for 6 h and 24 h, the expression of CD11b protein in the cerebral cortex of rats increased compared with that in the control group (*P* < 0.05), and in the EMP 6 h group, it increased significantly (*P* < 0.01) (Figure 3).

### 3.4. Effects of the Expression of TLR4 and NF*κ*Bp65 Induced by EMP in the Rat Cerebral Cortex

**Figure 4:**
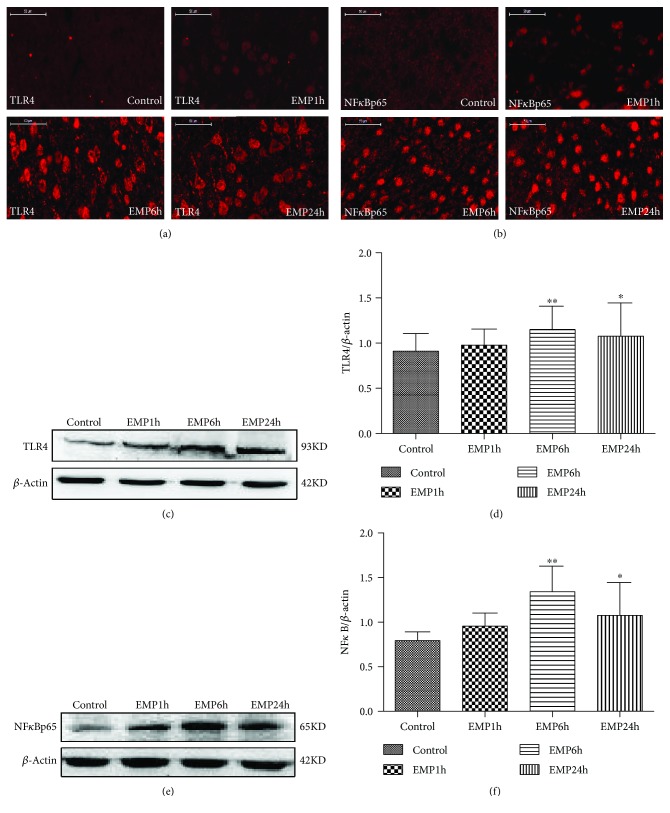
Effects of EMP on the TLR4/NF*κ*B signaling pathway expression in the rat cerebral cortex (*n* = 8). (a, b) Images showing the distributions and expression of TLR4 and NF*κ*Bp65 (red) immunoreactive cells in the cerebral frontal cortex for every group (scale bar = 50 *μ*m). (c–f) Effects of EMP exposure on TLR4 and NF*κ*Bp65 protein levels in the cerebral frontal cortex. The gels/blots were cropped to display the figures better. The data are presented as the means ± SEM. ^∗^ *P* < 0.05 vs. the control group and ^∗∗^ *P* < 0.01 vs. the control group.

The expression of TLR4 and NF*κ*Bp65 in the cerebral frontal cortex of rats after EMP exposure was detected by Western blotting and immunofluorescence (IF). The results showed that after EMP exposure, TLR4 and NF*κ*Bp65 protein expression was upregulated in the EMP6h group and EMP24h group compared with that in the control group (*P* < 0.05), the EMP6h group increased obviously (*P* < 0.01) (Figure 4).

### 3.5. Isoflurane Preconditioning Alleviates Neuronal Damage Induced by EMP

**Figure 5:**
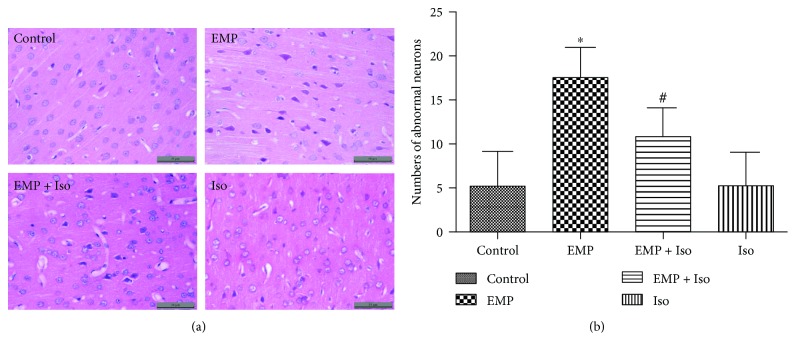
(a) HE staining in the frontal cortex of the rat brain (scale bar = 50 *μ*m). (b) Statistical graph of the number of abnormal neurons (^∗^ *P* < 0.05 vs. the control group and ^#^ *P* < 0.05 vs. the EMP group).

After EMP exposure (EMP group), the neurons were arranged in disorder, the cell body was sequestrated, the nucleus and the cytoplasm were dyed, the number of abnormal neurons increased, and the difference was statistically significant compared with the control group (control group; *P* < 0.05). After isoflurane preconditioning (EMP + Iso group), the number of abnormal neurons decreased compared with that in the EMP group (*P* < 0.05). The number of abnormal neurons in the frontal cortex of the isoflurane preconditioning group (Iso group) was not significantly different from that of the control group (Figure 5).

### 3.6. Isoflurane Preconditioning Alleviates the Inflammatory Response Induced by EMP

**Figure 6:**
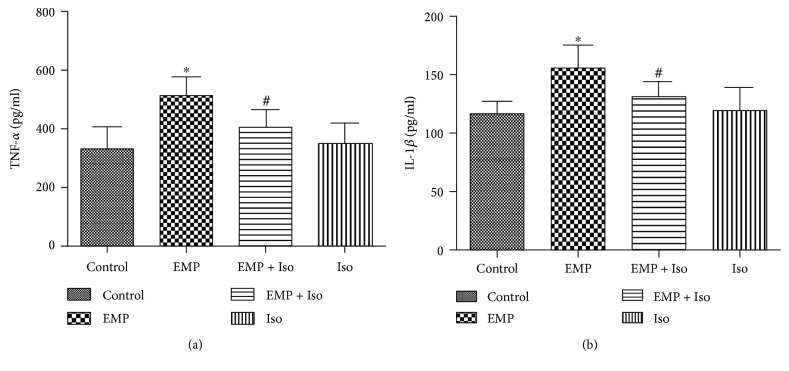
(a) The secretion level of TNF-*α*. (b) The secretion level of IL-1*β* (data are presented as the means ± SEM; ^∗^ *P* < 0.05 vs. the control group and ^#^ *P* < 0.05 vs. the EMP group).

ELISA was used to detect the secretion of proinflammatory cytokines TNF-*α* and IL-1*β* in the frontal cortex of the rats. The secretion level of TNF-*α* and IL-1*β* increased significantly in the EMP group compared to that in the control group (*P* < 0.05). In contrast, before EMP exposure, isoflurane preconditioning significantly decreased the secretion of proinflammatory cytokines TNF-*α* and IL-1*β* compared to that in the EMP group (*P* < 0.05). There was no significant difference in the secretion of TNF-*α* and IL-1*β* in the frontal cortex of the Iso group compared with that of the control group (*P* > 0.05) (Figure 6).

### 3.7. Isoflurane Preconditioning Alleviates the Microglia Activation Induced by EMP

**Figure 7:**
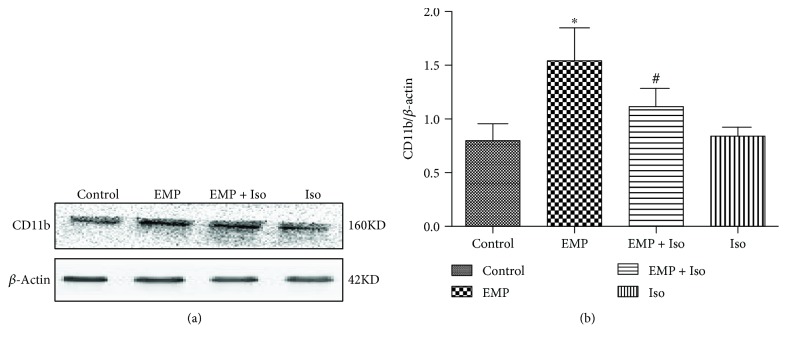
(a) Western blot of active CD11b. (b) Quantification of relative changes in active CD11b expression (data are presented as the means ± SEM; ^∗^ *P* < 0.05 vs. the control group and ^#^ *P* < 0.05 vs. the EMP group).

The activation marker protein CD11b of microglia was detected by Western blotting. The expression of CD11b increased significantly in the EMP group compared to that in the control group (*P* < 0.05). In contrast, before EMP exposure, isoflurane preconditioning significantly decreased the expression of CD11b compared to that in the EMP group (*P* < 0.05). There was no significant difference in CD11b in the frontal cortex of the Iso group compared with that of the Control group (*P* > 0.05) (Figure 7).

### 3.8. Isoflurane Preconditioning Alleviates Neuronal Apoptosis Induced by EMP

**Figure 8:**
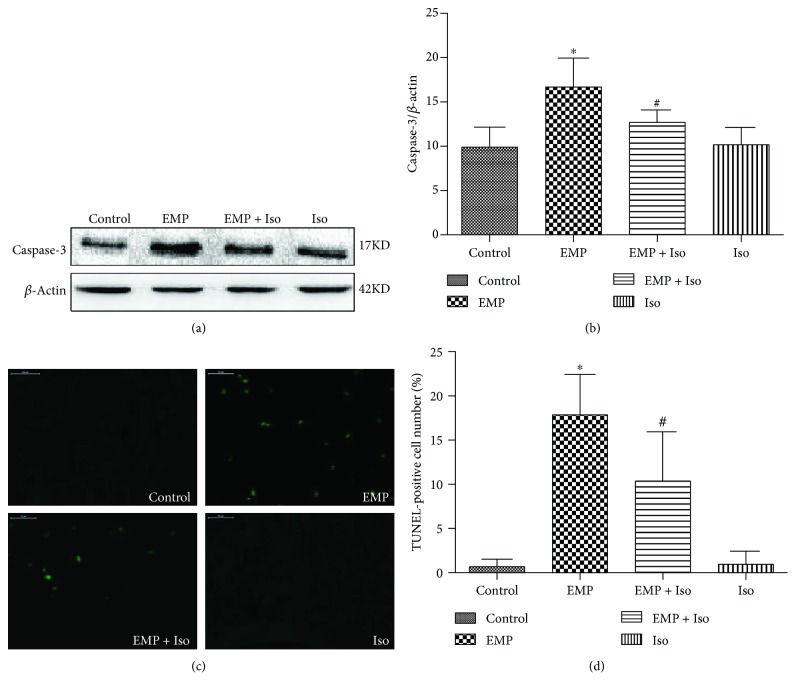
(a) Western blot of active caspase-3. (b) Quantification of relative changes in active caspase-3 expression. (c) Representative TUNEL staining of cerebral cortical neurons (scale bar = 50 *μ*m). (d) Number of TUNEL-positive cells in each group (data are presented as the means ± SEM; ^∗^ *P* < 0.05 vs. the control group and ^#^ *P* < 0.05 vs. the EMP group).

Caspase-3 expression in the cerebral frontal cortex of the brain was detected by Western blotting. The expression of caspase-3 increased significantly in the EMP group compared to that in the control group (*P* < 0.05). In contrast, before EMP exposure, isoflurane preconditioning significantly decreased the expression of caspase-3 compared to that in the EMP group (*P* < 0.05). There was no significant difference in caspase-3 in the frontal cortex of the Iso group compared with that of the control group (*P* > 0.05). The number of TUNEL-positive cells in the cerebral frontal cortex in the EMP group was greater than that in the control group (*P* < 0.05). Nevertheless, the number of TUNEL-positive cells in the EMP + Iso group was less than that in the EMP group (*P* < 0.05) (Figure 8).

### 3.9. Effects of Isoflurane Preconditioning on the Expression of the TLR4/NF*κ*B Signaling Pathway in the Frontal Cortex of the Brain after EMP Exposure

**Figure 9:**
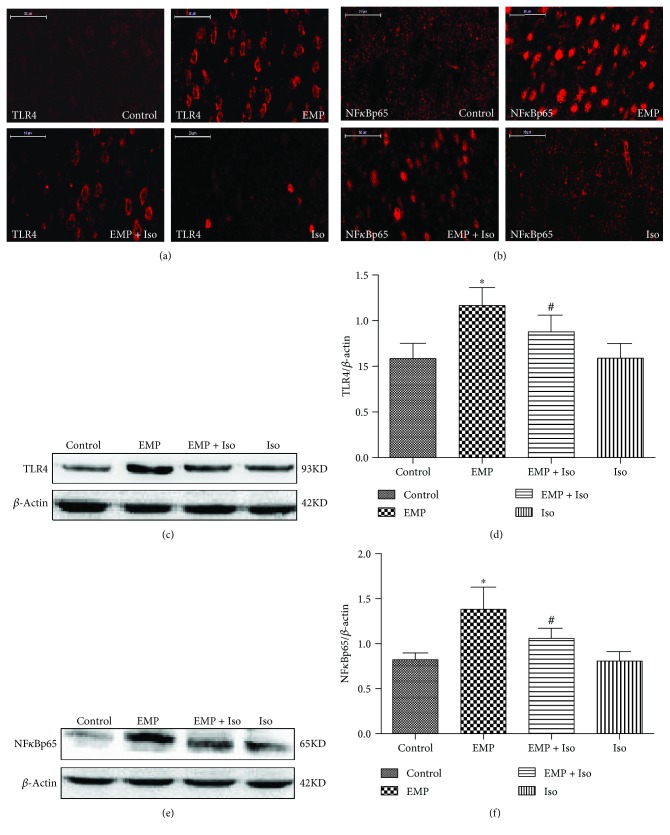
Effects of EMP on the TLR4/NF*κ*B signaling pathway expression in the rat cerebral cortex (*n* = 8). (a, b) Images showing the distribution and expression of TLR4 and NF*κ*Bp65 (red) immunoreactive cells in the cerebral frontal cortex for every group (scale bars = 50 *μ*m). (c–f) Effects of EMP exposure on TLR4 and NF*κ*Bp65 protein levels in the cerebral frontal cortex after EMP exposure. The gels/blots were cropped to better display the figures (data are presented as the means ± SEM; ^∗^ *P* < 0.05 vs. the control group and ^#^ *P* < 0.05 vs. the EMP group).

The expression of TLR4 and NF*κ*Bp65 in the cerebral frontal cortex of rats after EMP exposure was detected by Western blotting and immunofluorescence (IF). The results showed that after EMP exposure, TLR4 and NF*κ*Bp65 protein expression was upregulated compared with that in the control group (*P* < 0.05). In contrast, before EMP exposure, isoflurane preconditioning significantly decreased the expression of TLR4 and NF*κ*Bp65 compared to that in the EMP group (*P* < 0.05). There was no significant difference in the expression of TLR4 and NF*κ*Bp65 in the Iso group compared with that in the control group (*P* > 0.05) (Figure 9).

### 3.10. Isoflurane Preconditioning Improves Cognitive Function in Rats Exposed to EMP Radiation

**Figure 10:**
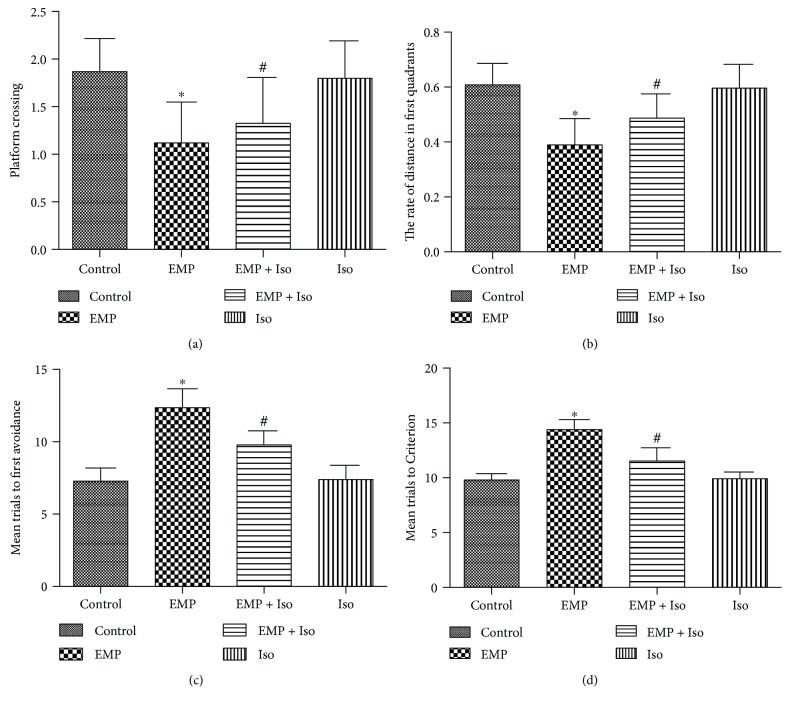
(a) Escape latency in the Morris water-maze test. (b) Rate of distance in the first quadrants of the Morris water-maze test. (c) Mean trials until first demonstration of avoidance on the T-maze. (d) Mean trials to criterion on the T-maze. ^∗^ *P* < 0.05 vs. the control group and ^#^ *P* < 0.05 vs. the EMP group.

The water-maze results show that the rats in the EMP group spent less time in the target quadrant (Figure 10(a)) than did rats in the control group (*P* < 0.05); however, after isoflurane preconditioning, this time period was longer than that in the EMP group (*P* < 0.05). The number of platform-site crossovers (Figure 10(b)) in the EMP group was lower than that in the control group (*P* < 0.05); however, the number of crossovers was greater in the EMP + Iso group than that in the EMP group (*P* < 0.05). These results suggest that EMP exposure impaired the learning and memory of rats, whereas isoflurane preconditioning alleviated this learning and memory damage.

The rats were also tested using the T-maze. The EMP group required more trials before the first demonstration of avoidance than did the control group (*P* < 0.05). However, the first demonstration of avoidance during acquisition showed that the EMP + Iso group required fewer trials to first demonstrate avoidance than did the EMP group (Figure 10(c)). Regarding trials to criterion on the retention test, the EMP group required significantly more trials than did the control group (*P* < 0.05), and the EMP + Iso group required fewer trials than did the EMP group (*P* < 0.05) (Figure 10(d)).

## 4. Discussion

The effects of electromagnetic radiation on human health cannot be ignored, and there have been many studies on the topic in recent years [20]. After SD pregnant rats are exposed to 900 MHz EMF, the subsequent learning and memory ability of female mice is affected, and histopathological changes are seen in the hippocampus [21]. Recently, some scholars have placed microglia (N9) and astrocytes in 1800-MHz RF conditions and measured the proinflammatory cytokine IL-1*β*, TNF-*α*, and the expression of mRNA; the results showed that the IL-1*β* mRNA level in microglia after an RF exposure of 12 h to 24 h increased significantly, TNF-*α* mRNA level in microglia after RF exposure of 3 h, 6 h, 12 h, and 24 h were increased, while this phenomenon was not found in astrocytes; additionally, RF-EMF induces a microglial inflammatory reaction by increasing the production of proinflammatory cytokines [22]. EMP is a unique type of electromagnetic radiation that causes changes in blood-brain barrier permeability. Rats that were exposed to an electromagnetic pulse irradiated by a field strength of 200 kv/m showed albumin exudate around the rat brain microvasculature, especially after 3 h of exposure [23]. EMP with the same field strength can also cause the activation of rat cerebral cortex microglia and induce an increase in TNF-*α* and IL-10, reaching a peak value after 6 h of exposure and returning to normal levels after 24 h [5]. A preliminary group study demonstrated that exposure to 400 kv/m EMP induced cerebral cortical neuronal damage and degeneration, apoptosis, and cognitive impairment [6]. Our early research essentially obtained the same result; after EMP exposure (400 kv/m), cortical neurons were damaged and induced cell apoptosis, and after 6 h of EMP exposure, microglia began to activate, resulting in the increased secretion of TNF-*α* and IL-1*β*, inducing an inflammatory reaction in the cerebral cortex.

Microglia have many functions, including supporting CNS development and synaptogenesis, maintaining homeostasis, generating immune responses to infection sources, and participating in neuroinflammation, neurodegenerative diseases, stroke, and trauma [24]. When pathological damage occurs, the microglia stimulated by endogenous or exogenous stimuli enter an active state. Similar to macrophages, the activation of microglia changes the shape of cells to exert phagocytosis, induce inflammatory responses, and release various cytokines and mediators to change the microenvironment homeostasis. As a result, the activity of microglia largely determines the fate of the other nerve cells around them [25–28]. Therefore, we conclude that EMP exposure induced the activation of microglia and released proinflammatory factor TNF-*α* and IL-1*β*, inducing inflammation and leading to neuronal apoptosis and injury. However, the signal transduction mechanism of EMP-induced microglia activation remains clear, and further study is needed on the topic.

We found that EMP activates the TLR4/NF*κ*B signaling pathway and the expression of TLR4 and NF*κ*Bp65 in the cerebral cortex of rats increases after EMP exposure. In the CNS, TLR4 is mainly expressed by glial cells, especially microglia [29]; TLR4 is also expressed in astrocytes, vascular endothelial cells, and neurons [16, 30]. Whether TLR4 is expressed in normal neurons is controversial [29], but the expression of TLR4 has essentially been confirmed in the pathological condition of neurons [30, 31]. TLR4 plays an important role in cerebral ischemia-reperfusion injury; TLR4 expression was increased in cerebral cortical neurons in response to ischemia/reperfusion injury [30]. In the astrocytes of the spinal cord, TLR4 activation leads to cascade amplification, which leads to the activation of NF*κ*B. Subsequently, an increase is seen in proinflammatory cytokines and stress response mediators, such as TNF-*α*, COX-2, and iNOS, all of which are related to CNS diseases [17–19]. In the microglia, the TLR4/NF*κ*B signaling pathway shows the same results. After cerebral ischemia-reperfusion injury, TLR4 expression is increased, resulting in a decrease in I*κ*B expression, the activation of NF*κ*B and microglia, and the increased expression of related proapoptotic proteins [14]. Subedi et al. [32] found a neuroprotective effect of equol in vitro by using LPS to simulate the brain's inflammatory reaction; LPS can increase the expression of the TLR4 protein and NF*κ*B activation, leading to the activation of microglia, inducing the release of proinflammatory cytokine TNF-*α*, and aggravating inflammatory reactions. Therefore, we deduce from the experimental results that the mechanism of the EMP-induced neurotoxicity of microglia activation may be related to the TLR4/NF*κ*B signaling pathway.

Research on protection against electromagnetic pulse has mainly focused on physical protection, such as new wave absorbing materials and plasma protection technology. However, few studies have been performed to examine methods of drug protection. In this experiment, electromagnetic pulse drug protection was studied. Most studies have shown that isoflurane preconditioning improves cerebral infarct volume in adult male rodents with transient or permanent focal cerebral ischemia [33–37]. The number of TUNEL-positive neurons in the CA1 region of the hippocampus was found to decrease in 1.2% isoflurane preconditioning, indicating that this preconditioning could improve the survival of the neurons in the CA1 region of the whole brain ischemic hippocampus [38]. We found that isoflurane preconditioning could also reduce nerve damage caused by EMP. The experimental results show that before EMP exposure, isoflurane preconditioning treatment can decrease the number of abnormal neurons in the prefrontal cortex, the activity of caspase-3, and neuronal apoptosis and reduce the levels of proinflammatory factors TNF-*α* and IL-1*β*. This finding suggests that isoflurane preconditioning has a neuroprotective effect against EMP. Meanwhile, our findings suggest that isoflurane preconditioning can improve learning and memory impairment induced by EMP in rats. Isoflurane preconditioning exerts neuroprotective effects through multiple mechanisms; these studies of isoflurane preconditioning neuroprotective mechanisms focus on the inhibition of microglia activation, the downregulation of the TLR4/NF*κ*B signaling pathway, and the reduction of the inflammatory response. Research has found that 2% isoflurane preconditioning provides neuroprotection by directly regulating the TLR4 signaling pathway and alleviating microglial activation in the ischemic penumbra [14]. When isoflurane preconditioning was performed before EMP exposure, the expression of TLR4 and NF*κ*Bp65 in the cerebral frontal cortex was downregulated. This finding indicated that isoflurane preconditioning could reduce brain damage caused by electromagnetic pulse, and it may reduce the expression level of TLR4 and NF*κ*Bp65, affect the TLR4 signaling pathway, and inhibit the activation of NF*κ*B, thereby inhibiting the occurrence of inflammatory reactions.

## 5. Conclusions

In summary, we confirmed that exposure to 400 kv/m EMP induced cerebral cortical neuronal damage and apoptosis, and we found that the mechanism involves the TLR4/NF*κ*B signaling pathway, activating microglia and inducing an inflammatory reaction; however, isoflurane preconditioning conferred protective effects against EMP-induced brain injury by regulating the TLR4/NF*κ*B signaling pathway and alleviating microglial activation. This study provides a new therapeutic target for electromagnetic pulse damage and a novel experimental basis and pharmacological direction for the protection against and treatment of electromagnetic pulse.

## Data Availability

The data used to support the findings of this study are available from the corresponding author upon request.

## Supplementary Materials

Supplementary Materials Supplementary Figure 1: effects of EMP on the expression of TLR4 in the microglia and neuron. (a) TLR4 (green) with microglial-specific marker (Iba-1; red) for every group. (b) TLR4 (green) with neuronal-specific marker neuronal nuclei (NeuN; red) for every group. Scale bars = 50 *μ*m. Supplementary Figure 2: effects of EMP on the morphology of microglia in the rat cerebral cortex. Images showing the microglial-specific marker (Iba-1; red) in the cerebral frontal cortex for every group. Scale bars = 50 *μ*m.
