# Supplementary material for: Isoflurane Preconditioning Attenuates Brain Injury Induced by Electromagnetic Pulse via the TLR4/NFκB Signaling Pathway
Source: Oxid Med Cell Longev. 2019 Jan 6;2019:9653494. doi: 10.1155/2019/9653494 (PMC6339739; doi:10.1155/2019/9653494)
Supplement: Supplementary Materials — Supplementary Figure 1: effects of EMP on the expression of TLR4 in the microglia and neuron. (a) TLR4 (green) with microglial-specific marker (Iba-1; red) for every group. (b) TLR4 (green) with neuronal-specific marker neuronal nuclei (NeuN; red) for every group. Scale bars = 50 μm. Supplementary Figure 2: effects of EMP on the morphology of microglia in the rat cerebral cortex. Images showing the microglial-specific marker (Iba-1; red) in the cerebral frontal cortex for every group. Scale bars = 50 μm. [file 9653494.f1.pdf]

Supplementary Materials

Effects of EMP on the expression of TLR4 in the microglia and neuron.

SD rats were randomly divided into 4 groups: Control group, 6 h after EMP group (EMP group), EMP exposure after isoflurane preconditioning group (EMP+Iso group), and isoflurane preconditioning group (Iso group). We performed double immunofluorescent staining of TLR4 (green) with microglial specific marker (Iba-1; red) and TLR4 (green) with neuronal-specific marker neuronal nuclei (NeuN; red). The results showed that TLR4 expressed in microglia after EMP exposure. Before EMP exposure, isoflurane preconditioning decreased the activation degree of microglia and expression of TLR4. There was no difference in the expression of TLR4 and Iba1 in the Iso group compared with that in the Control group (Figure 1a). We also found that TLR4 was increased in neurons after EMP exposure. Before EMP exposure, isoflurane preconditioning decreased the expression of TLR4. There was no difference in the expression of TLR4 in neurons in the Iso group compared with that in the Control group (figure 1b).

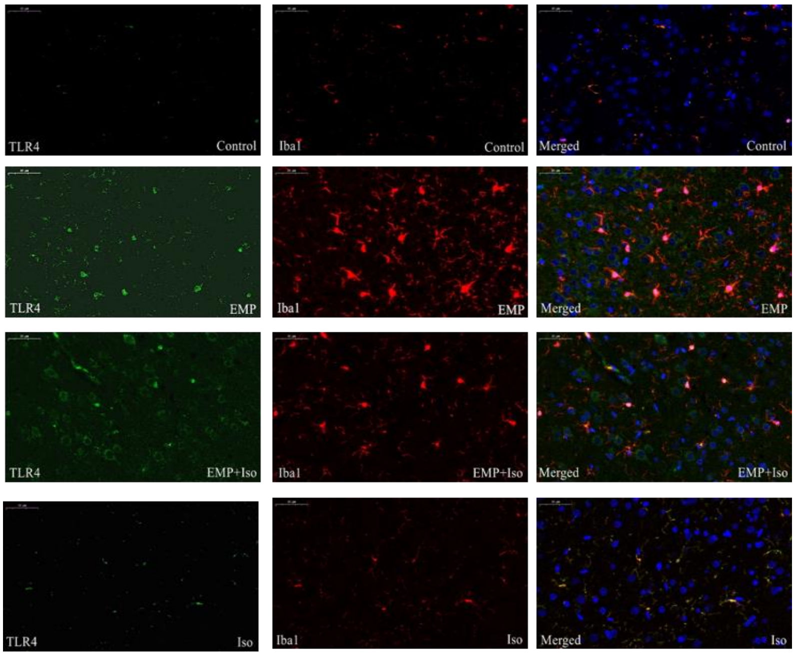

(a)

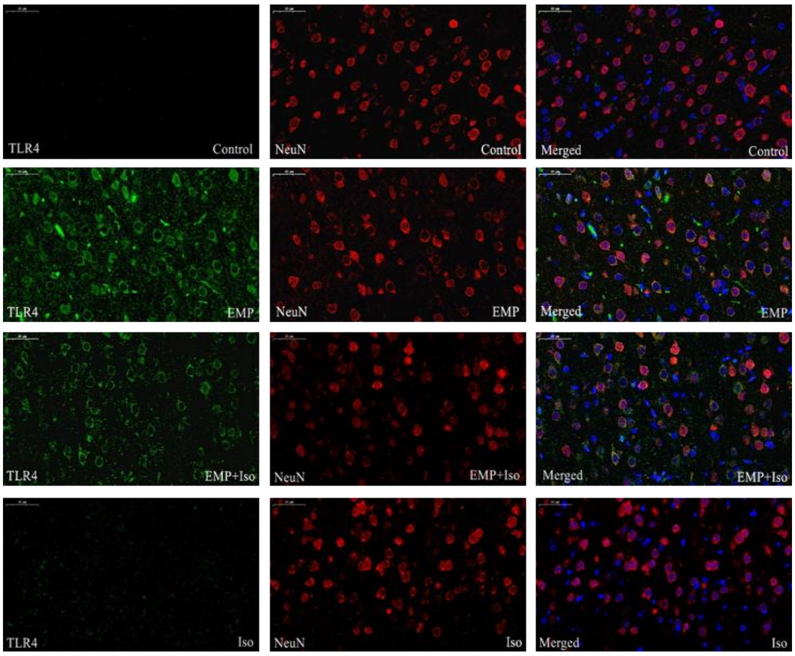

(b)

Figure 1: Effects of EMP on the expression of TLR4 in the microglia and neuron. (a) TLR4 (green) with microglial specific marker (Iba-1; red) for every group. (b) TLR4 (green) with neuronal-specific marker neuronal nuclei (NeuN; red) for every group. (Scale bars=50 μm).

Effects of EMP on the morphology of microglia in the rat cerebral cortex.

The microglia was identified using microglial specific marker Iba1 (red) labelling (with DAPI to show the nucleus). The results showed that in the control group and isoflurane preconditioning group, the microglia had smaller cell bodies, more and finer synapses, and most of the microglia were in a resting state. After EMP exposure, the microglia changed from resting branching to circular amoebic, the cell bodies increased and branching decreased. In contrast, before EMP exposure, isoflurane preconditioning significantly decreased the activation degree of microglia, and compared with EMP group, activated microglia cell body had decreased (Figure 2).

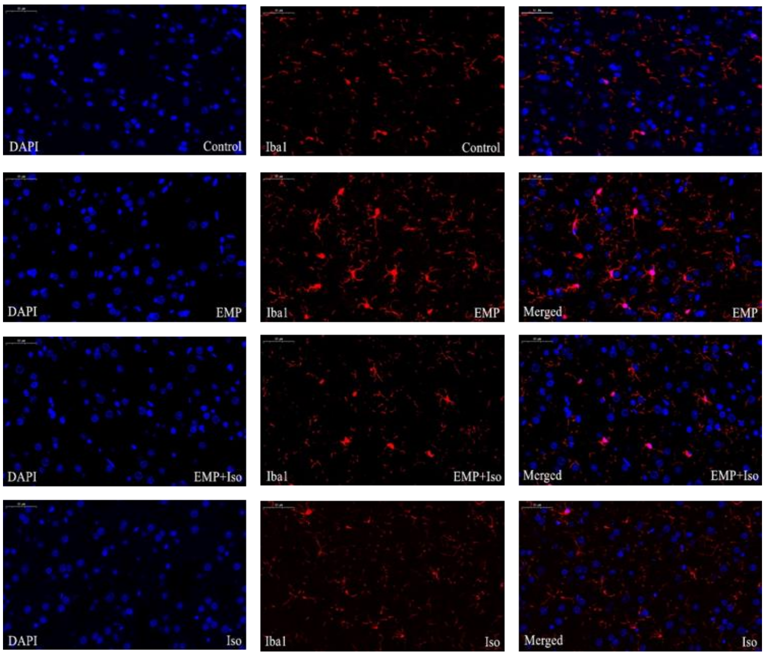

Figure 2: Effects of EMP on the morphology of microglia in the rat cerebral cortex. Images showing the microglial specific marker Iba1 (red) in the cerebral frontal cortex for every group. (Scale bars=50 μm).
